# Eco-House Prototype Constructed with Alkali-Activated Blocks: Material Production, Characterization, Design, Construction, and Environmental Impact

**DOI:** 10.3390/ma14051275

**Published:** 2021-03-08

**Authors:** Rafael A. Robayo-Salazar, William Valencia-Saavedra, Sandra Ramírez-Benavides, Ruby Mejía de Gutiérrez, Armando Orobio

**Affiliations:** 1 Composites Materials Group (CENM), School of Materials Engineering, Universidad del Valle, Calle 13 #100-00, E44, Cali 76001, Colombia; rafael.robayo@correounivalle.edu.co (R.A.R.-S.); william.gustavo.valencia@correounivalle.edu.co (W.V.-S.); sandra.ramirez.benavides@correounivalle.edu.co (S.R.-B.); 2 Applied Research in Construction Group (GRUA), School of Civil Engineering, Universidad del Valle, Calle 13 #100-00, Cali 76001, Colombia; armando.orobio@correounivalle.edu.co

**Keywords:** alkali-activated material, geopolymer, concrete block, brick, eco-friendly house, sustainable construction, waste recycling

## Abstract

The interest of the construction industry in alkali-activated materials has increased to the extent that these materials are recognized as alternatives to ordinary Portland cement-based materials in the quest for sustainable construction. This article presents the design and construction of a prototype of an eco-friendly house built from concrete blocks produced using alkali activation technology or geopolymerization. The prototype meets the requirements of the current Colombian Regulations for Earthquake Resistant Buildings (NSR-10) and includes standards related to the performance of the materials, design, and construction method for earthquake-resistant confined masonry of one- or two-story buildings. The alkali-activated blocks were obtained from different precursors (aluminosilicates), including a natural volcanic pozzolan, ground granulated blast furnace slag, fly ash, construction and demolition waste (concrete, ceramic, brick, and mortar), and red clay brick waste. The physical-mechanical characterization of the alkali-activated blocks allowed their classification according to the structural specifications of the Colombian Technical Standard NTC 4026 (equivalent to ASTM C90). The global warming potential (GWP) or “carbon footprint” attributed to the raw materials of alkali-activated blocks was lower (25.4–54.7%) than that of the reference blocks (ordinary Portland cement concrete blocks). These results demonstrate the potential of alkali-activated materials for application in the construction of eco-friendly houses.

## 1. Introduction

The search for emerging technologies that promote environmental sustainability is a priority for the construction sector; this sector is among the top contributors in global pollution indices. This problem has been identified in the framework of the “17 Sustainable Development Goals” of the “2030 Agenda for Sustainable Development of the United Nations General Assembly”. According to the United Nations (UN), rapid urbanization, promoted by exponential population growth, will cause a 60% increase in housing demand by 2030, making cities the generators of 75% of the world’s global carbon emissions. Likewise, by 2050, an increase in natural resources equivalent to almost three planets-worth is estimated to be necessary to sustain current lifestyles. For these reasons and others, the approach and urgent implementation of the model called “sustainable cities” by UN member countries have been proposed [1]. This model proposes sustainable consumption and production, seeking to decouple economic growth from environmental degradation, to increase resource efficiency, to reduce the extraction of raw materials and the final disposal of the waste generated (“zero waste” approach), and to move towards green (circular) economies with low carbon emissions [2].

In recent decades, the technology of alkaline activation or geopolymerization has caught the attention of the scientific and construction sectors, mainly due to the possibility of alternatives to ordinary Portland cement (OPC)-based materials with a lower “carbon footprint” or global warming potential (GWP) and with superior mechanical and durability performances [3,4,5,6], with life cycle analysis (LCA) regaining importance [7,8,9]. Additionally, alkali-activated technology facilitates the use of various industrial wastes or by-products such as aluminosilicate materials in obtaining alkali-activated materials and geopolymers. These materials are obtained from the relatively low-temperature (25–100 °C) physicochemical interaction between aluminosilicates (precursors) and strongly alkaline solutions (alkaline activators), which results in precipitation of reaction products: sodium aluminosilicate hydrate (N-A-S-H) gels (poor in calcium) and/or “hybrid” C-(N)-A-S-H gels (rich in calcium), with excellent cementing and binding characteristics. These alkali-activated materials can be used in various products, such as mortars, concrete, and construction elements (prefabricated) such as bricks and concrete blocks [10,11].

The use of alkali-activated materials in the production of masonry blocks and bricks has been validated by several authors, such as the reviews by Gavali et al. [12] and Deraman et al. [13], with fly ash (FA) [14,15,16] being the most common primary precursor in these studies. Indeed, Ariöz et al. [17] produced geopolymeric bricks with compressive strengths between 5 and 60 MPa from the alkaline activation (NaOH + Na_2_SiO_3_) of FA by optimizing the heat curing process at 60 °C for 24 h. Gavali and Ralegaonkar [18] reported the production of solid bricks from the alkaline activation (NaOH + Na_2_SiO_3_) of a mixture composed of 80% rice husk ash and 20% FA, with stone dust as a filler (precursor/filler ratios of 1:1, 1:2, and 1:3 (by weight)). The bricks reached compressive strengths (28 days) between 5 and 15 MPa and absorption values between 6 and 14%. In a complementary study, Gavali and Ralegaonkar [19] proposed the design of an eco-house from these alkali-activated bricks. However, their construction process was not reported. Huynh et al. [20] and Hwang and Huynh [21] highlighted the possibility of substituting FA for rice husk ash (10–50%) in alkali-activated blocks (NaOH), reporting an optimal substitution rate of 10%, in agreement with the results reported by Maulana et al. [22]. Poinot et al. [23] were able to obtain alkali-activated bricks (NaOH) from a mixture composed of 70% FA, 20% clay, and 10% hydrated lime (as a source of calcium) with strengths between 11 and 15 MPa at 28 days. Venugopal et al. [24] manufactured alkali-activated bricks (solid and hollow) from a mixture of FA (80%) and granulated blast furnace slag (GBFS) (20%) (as a source of calcium). In these mixtures, NaOH and Na_2_SiO_3_ were used as alkaline activators and fine aggregates with an FA–GBFS/sand ratio of 1:1 (by weight). The physical-mechanical characterization of the bricks yielded compressive strengths between 5 (3 days) and 25 (28 days) MPa and absorption values (28 days) between 8.2 and 9.1%. Mohammed et al. [25] reported the production of geopolymer hollow bricks based on the activation of FA with mixtures of NaOH and Na_2_SiO_3_ and crumb rubber as aggregates (FA/rubber ratio 1:1 (by weight)), reaching a compressive strength (28 days) of 3.98 MPa and an absorption value of 25.2%.

Another by-product used as a primary precursor or source of calcium (addition) in the production of alkali-activated bricks or blocks is GBFS. Indeed, Ren et al. [26] reported the synthesis and physical-mechanical characterization of alkali-activated blocks (NaOH + Na_2_SiO_3_) based on GBFS, with partial substitutions of waste concrete powder (20, 40, and 60% by weight) and a recycled fine aggregate (0, 25, 50, 75, and 100% by weight). The results of the compressive strength showed values between 15 and 60 MPa depending on the content of concrete powder and recycled fine aggregate, with absorption values ranging between 13 and 25%. Ahmari and Zhang [27,28,29] demonstrated the potential of using copper mine tailings in the production of alkali-activated bricks (NaOH) with compressive strengths of up to 15 MPa and absorption values lower than 5%, which were achieved by optimizing the compaction pressure and the curing temperature. Chen et al. [30] used a bottom ash from circulating fluidized bed combustion as a precursor of alkali-activated geopolymer bricks with solutions of NaOH and Na_2_SiO_3_, reaching compressive strength values of 16.1 and 21.9 MPa at 7 and 28 days of curing, respectively.

All these results demonstrate, from the technological and physical-mechanical point of view, the possibility of using alkaline activation technology in the production of alkali-activated bricks or blocks. Regarding the potential of reducing the “carbon footprint” (environmental impact) related to this application, some authors reported promising results. Apithanyasai et al. [31] evaluated the potential of using mixtures of waste foundry sand, FA, and electric arc furnace slag in proportions of 70:30:0, 60:30:10, 50:30:20, and 40:30:30% (by weight) as precursors of geopolymer bricks, activated with NaOH and Na_2_SiO_3_ solutions. The optimal mixture corresponding to a ratio of 40:30:30 yielded a compressive strength of 25.8 MPa. The authors highlighted that this alkali-activated brick has a lower environmental impact than OPC concrete blocks. Likewise, Dahmen et al. [32] performed LCA on blocks based on the alkaline activation (NaOH + Na_2_SiO_3_) of kaolinite clays, showing that this type of block has a carbon footprint (GWP) of 1.03 kg∙CO_2_∙eq/block, which is 41.5% more environmentally friendly than that of an OPC concrete block (1.76 kg∙CO_2_∙eq/block).

This article is an addition to the growing number of publications related to the development of alkali-activated materials, highlighting their application potential in the production of environmentally friendly structural masonry blocks suitable for the construction of one- and two-story buildings that meet all structural and earthquake resistance specifications. Among the raw materials, various types of precursors (aluminosilicates) were used, such as FA, GBFS, natural pozzolan (NP), construction and demolition waste (concrete, ceramic, mortar, and brick wastes) (CDW), and fired red clay brick waste (chamotte) (RCBW). Additionally, recycled aggregates (fine and coarse) from CDW were used. This research has continuity with several studies [33,34,35,36,37,38,39] previously developed by the Composite Materials Group of the Universidad del Valle (Cali-Colombia) in the alkali activation and geopolymerization line of research [40]. In these previous studies, the contents of the alkaline activator (NaOH + SS + water) and precursors (NP–GBFS; FA; CDW; RCBW) of each geopolymeric mixture were defined.

This article includes (a) the selection and characterization of raw materials, (b) the production and physical-mechanical characterization of alkali-activated blocks, (c) the architectural-structural design and the construction of an eco-house prototype, and (d) the estimation of the environmental impact (carbon footprint) associated with the materials and transport of raw materials used in the production of alkali-activated blocks. It should be noted that, prior to this publication, there were no reports of the construction of houses from alkali-activated or geopolymeric blocks. The objective of this paper was to demonstrate the application potential of alkali-activated materials in the production of blocks for the construction of environmentally sustainable housing, complying with all the physical-mechanical specifications established by the technical standards and construction regulations that guarantee their structural performance and earthquake (or seismic) resistance in Colombia. Additionally, the eco-house prototype represents an interesting and high-potential object of future studies that will be related to thermal measurements (comfort) and long-term durability.

## 2. Materials and Methods

### 2.1. Raw Materials

The precursors (aluminosilicates) used for the production of the alkali-activated blocks, with which the eco-house prototype was built, were: (1) natural pozzolan (NP), (2) fly ash (FA), (3) construction and demolition waste (CDW), and (4) red clay brick waste (RCBW). The NP corresponds to a pozzolan of volcanic origin, from the central region of Colombia. The FA was collected from a brick company in the region (by-product of coal combustion). The CDW was composed of a mixture (25% by weight of each waste) of concrete, ceramic (red and white), mortar, and brick wastes, collected from the final disposal site of the city of Cali (Colombia). The RCBW corresponds to a sample of “chamotte” (by-product from industrial brick manufacturing) from a clay brick company in the region.

To promote curing at room temperature (25 °C) of the alkali-activated blocks, additions of calcium-rich materials were used; in the case of the NP, 30% of granulated blast furnace slag (GBFS) with respect to the weight of the NP was added. GBFS corresponds to a by-product of the steel industry. In the case of FA, CDW, and RCBW, 10% Portland cement (OPC) in relation to the weight of the precursor was added. For comparative purposes, the OPC was also used to produce reference concrete blocks. Table 1 presents the chemical composition of the raw materials used. The chemical composition was determined by X-ray fluorescence (XRF) using a Phillips PANalytical MagiX PRO PW 2440 spectrometer (Tollerton, UK) equipped with a rhodium tube, whose maximum power is 4 kW. The aluminosilicate nature (SiO_2_ + Al_2_O_3_ = 58–86%) of the precursors NP, FA, CDW, and RCBW is highlighted, as well as the high CaO content of GBFS (40.3%) and OPC (62.3%). Precursors were subjected to crushing and grinding processes until obtaining a particle size close to that of OPC (21.6 µm). Indeed, the average particle sizes of NP, GBFS, FA, CDW, and RCBW were 20.6, 26.4, 24.9, 92.1, and 24.2 µm, respectively; this analysis was performed by laser granulometry using a Mastersizer-2000 (Malvern Instruments equipment, Malvern, UK). The particle density of these powders ranged between 2396 and 2918 kg/m^3^. The OPC reported a higher density value (3121 kg/m^3^).

For the alkaline activation of the precursors, mixtures of sodium hydroxide (NaOH) and sodium silicate (SS) or “waterglass” (Na_2_SiO_3_: SiO_2_ = 32.09%, Na_2_O = 11.92%, H_2_O = 55.99%) were used. The optimal dosage of NaOH and SS set for each mixture was dissolved in the mixing water, thus obtaining the “alkaline activator solution”.

To produce the alkali-activated blocks, fine and coarse aggregates of natural and recycled origin were used. The recycled aggregates were obtained from the crushing of construction and demolition waste. The coarse recycled aggregate (CRA) was obtained from the crushing (25.4 mm) of concrete waste. To produce the fine recycled aggregate (FRA), ceramic waste (white and red) and mortar waste were crushed. The coarse natural aggregate (CNA) corresponds to a crushed siliceous gravel (maximum size = 25.4 mm) and the fine natural aggregate (FNA) to a siliceous sand extracted from a river in the region (Cali, Colombia). The main characteristics of the aggregates can be observed in Table 2.

The highest absorption percentage of the recycled aggregates is highlighted (CRA = 9.2% and FRA = 12.1%), related to their lower density, if compared with natural aggregates (CNA = 2% and FNA = 1.9%). The CRA reported a resistance to degradation by abrasion and impact in the Los Angeles Machine of 33.6%, higher than the 16.4% reported by the CNA. In general, the aggregates meet the specifications established in the ASTM C33 [41] standard to produce mortar–concrete mixtures.

### 2.2. Methodology

**Figure 1:**
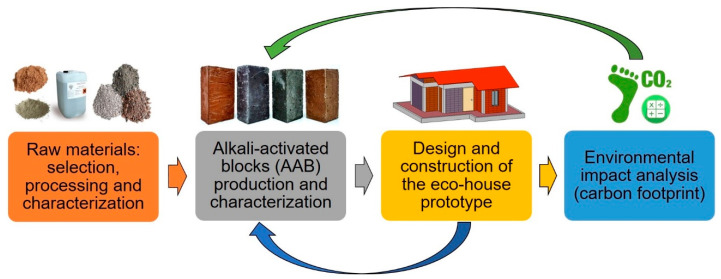
Schematic of the methodology used in this research.

Figure 1 presents a graphical abstract of the methodology used in this research, which includes the execution of four stages or phases: (1) the selection, characterization, and processing of the raw materials, (2) the production and characterization of the alkali-activated blocks, (3) the design and construction of the eco-house prototype, and (4) the analysis of the environmental impact (carbon footprint).

From the raw materials described in Section 2.1, the production of reference blocks (based 100% OPC) and alkali-activated blocks (NP–GBFS, FA, CDW, and RCBW) was carried out using alkaline activation or geopolymerization processes. In each case, the precursors (NP, FA, CDW, and RCBW) were dry mixed with calcium additions (GBFS and OPC) and mechanically homogenized in a horizontal mixer (CreteAngle planetary mixer, Ferring, UK). Subsequently, the alkaline activator (NaOH + SS + mixing water) was added to the mixture, yielding an alkali-activated paste. Once the paste was obtained, the aggregates (fine and coarse), previously homogenized and pre-saturated, were incorporated. The conventional mixing method of dry homogenizing the OPC and the aggregates (fine and coarse) prior to incorporating the mixing water was used to produce the reference blocks (100% OPC). The total mixing time was 10 min. The mixtures were cast into molds of 10 rectangular cavities and subjected to a manual compaction process (three layers) and mechanical vibration (30 s) to remove the air naturally trapped in the mixture. Prior to hardening, surface finishing of the mixtures was applied. The molds were covered with a plastic film for the first 24 h and held at room temperature (25 °C). Subsequently, they were demolded and held in a curing chamber at 25 °C and a relative humidity greater than 80% for 28 days.

The mixtures were mechanically characterized by compressive strength tests, after 7 and 28 days of curing. The compressive strength of the mixtures was evaluated according to the procedure described in the ASTM C39 standard [47], using standard 76.2 mm diameter cylinders. After the production of the alkali-activated blocks, these were also physically-mechanically characterized according to the requirements established by the Colombian Technical Standard (NTC) 4026 [48] (equivalent to the ASTM C90 [49] standard), which allowed their classification according to performance. The characterization tests were carried out at the age of 28 days of curing and included the determination of the compressive strength, the modulus of rupture by flexure, and the density, absorption, and porosity. The mechanical tests were carried out in an ELE International hydraulic press with 1000 kN capacity. The results reported in the physical-mechanical tests correspond to the average of three specimens.

The design and construction of the eco-house prototype was made from alkali-activated blocks, following the specifications established in the Colombian Regulations for Earthquake Resistant Construction (NSR-10) [50], including standards related to the performance of materials, design, and construction method for one- and two-story earthquake-resistant buildings of confined masonry.

In order to determine the environmental sustainability of the eco-house prototype, the global warming potential (GWP) or “carbon footprint” associated with the mixtures used for the production of the alkali-activated blocks (NP–GBFS, FA, CDW, and RCBW) was calculated and compared with that reported by the reference blocks based 100% on OPC. LCA in accordance with ISO 14,040 [51] included: (1) definition of the objective and scope, (2) inventory analysis, (3) impact analysis, and (4) interpretation. In the present study, the system boundary for comparison of the different mixtures was “cradle to gate”; therefore, the mixing, molding, and curing processes were excluded considering that they were identical in all samples.

## 3. Results and Analysis

### 3.1. Mechanical Characterization of the Mixtures

Table 3 shows the proportions of the mixtures for each type of block, including the alkali-activated blocks (NP–GBFS, FA, CDW, and RCBW) and the reference blocks based on 100% OPC. The designs of these mixtures were based on the “absolute volume” methodology proposed by ACI 211.1 [52]. In the case of the alkali-activated mixtures, the contents of the alkaline activator (NaOH + SS + water) and precursors were selected based on previous studies (NP–GBFS [33,53]; FA [34]; CDW [38]; RCBW [37,54]).

**Figure 2:**
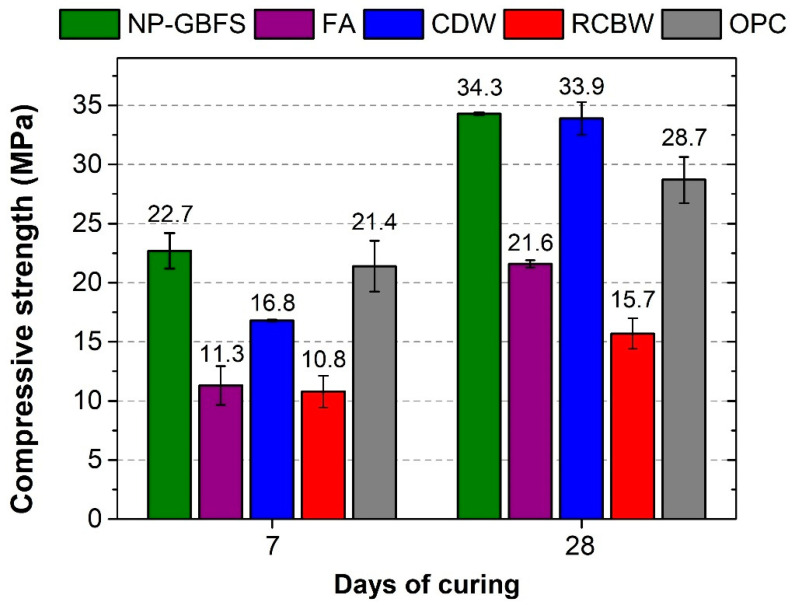
Compressive strength of the mixtures (7 and 28 days) (curing conditions: 25 °C, 80% relative humidity (RH)).

Figure 2 shows the compressive strengths (7 and 28 days) reached by the alkali-activated (NP–GBFS, FA, CDW, and RCBW) and reference (100% OPC) mixtures. The compressive strength values reported for the NP–GBFS, FA, CDW, and RCBW mixtures at 28 days of curing were 34.3, 21.6, 33.9, and 15.7 MPa, respectively, compared with the 28.7 MPa reached by the reference mixture based on 100% OPC. The high strength (33.9 MPa) of the CDW mixture was highlighted, taking into account that both the binder (90%) and the aggregates (100%) (fine and coarse) corresponded to CDW. In general, the alkali-activated mixtures exhibited a higher strength gain than the OPC mixture between 7 and 28 days, reporting 51.1% (NP–GBFS), 91.2% (FA), 101.8% (CDW), and 45.4% (RCBW) compared to the 34.1% for the OPC mixture. This finding highlights an important technological advantage of these materials; the strength of alkali-activated materials has been shown to increase over time [3,55] without the strict processes of wet curing or immersion in water that OPC-based (hydraulic) materials undergo to achieve this behavior [56].

NSR-10 [50] establishes, in equivalence to ACI 318 [57], that the minimum compressive strength from which a mixture is considered suitable for use in concrete structural elements is 17.5 MPa at 28 days, a value that meets three of the evaluated mixtures. However, in the case of precast elements, such as solid concrete blocks, the NTC 4026 [48] standard (equivalent to the ASTM C90 [49] standard) classifies these products based on their weight and the level of strength and absorption, as discussed in Section 3.3.

### 3.2. Alkali-Activated Blocks Production

**Figure 3:**
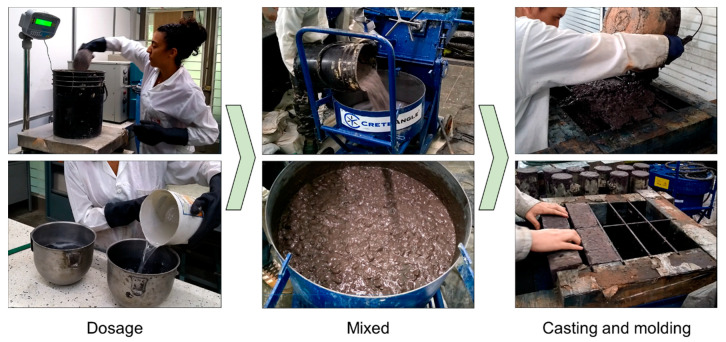
Blocks production process.

Figure 3 presents the production process of the alkali-activated blocks, which was based on the mixing proportions defined in Table 3.

**Figure 4:**
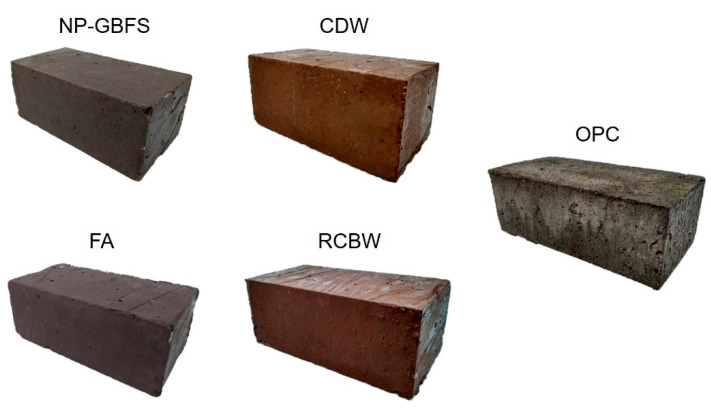
Blocks produced: alkali-activated (natural pozzolan–granulated blast furnace slag (NP–GBFS), fly ash (FA), construction and demolition waste (CDW), and red clay brick waste (RCBW)) and reference (OPC).

The solid concrete blocks produced correspond to rectangular units 20 cm long, 10 cm wide, and 8 cm thick (height), as shown in Figure 4. The construction of the eco-house prototype utilized a total of 964 prefabricated units, divided as follows: 67 units of NP–GBFS, 173 units of FA, 140 units of CDW, 255 units of RCBW, and 329 units of OPC (reference). In Section 3.4, the type of block per wall is defined according to the architectural design and the structural drawing of the eco-house prototype.

### 3.3. Physical-Mechanical Characterization of the Alkali-Activated Blocks

Table 4 lists the classification and requirements for the compressive strength and water absorption (according to weight) of the concrete units for structural masonry according to the NTC 4026 [48] standard (equivalent to ASTM C90 [49]), and the specifications established by NSR-10 [50] for use in the construction of earthquake-resistant one- or two-story houses in Colombia. Table 5 presents the results of the physical-mechanical characterization of the alkali-activated (NP–GBFS, FA, CDW, and RCBW) and reference (OPC) blocks.

In the case of the NP–GBFS blocks (Table 5), the compressive strength was 31.4 MPa, which exceeds the minimum strength (13 MPa) established by NTC 4026 [48] for blocks classified as “high-strength structural” masonry concrete by 141%. The maximum absorption allowed for this classification, considering the density of the NP–GBFS blocks (1902 kg/m^3^), is 12% (Table 4). Table 5 shows that the water absorption reported by the NP–GBFS block was 8.3%. The porosity and modulus of rupture (flexural strength) of the NP–GBFS block were 15.6% and 4.8 MPa, respectively.

The FA blocks (Table 5) yielded a compressive strength of 23.9 MPa, a value that exceeds the minimum strength (13 MPa) established by the NTC 4026 standard [48] for blocks classified as “high-strength structural” by 84% (Table 4). The absorption value was 7.5%, which is within the range (<9%) of this classification considering the density of the block (2295 kg/m^3^). Likewise, the FA blocks reached values of porosity and modulus of rupture (flexural strength) of 17.2% and 4.6 MPa, respectively. If these results are compared with those reported by other authors for alkali-activated blocks based on FA (see Introduction Section), it is found that, in general, the results are satisfactory. For example, the compressive strength of FA blocks (23.9 MPa) is superior to the results reported by Poinot et al. [23] (11–15 MPa) and Venugopal et al. [24] (5–25 MPa).

Note that for the CDW blocks (Table 5), although their compressive strength (26.1 MPa) was 101% higher than the minimum limit (13 MPa) established by the NTC standard 4026 [48] for the classification of a “high-strength structural” block, their water absorption (14.4%) exceeded the maximum value allowed (12%) for medium-weight blocks (density 1926 kg/m^3^). However, the CDW blocks could be classified as a “low-strength structural” blocks since the maximum tolerated absorption value for this classification is 15% (Table 4). The porosity and modulus of rupture (flexural strength) of the CDW blocks were 27.7% and 3.6 MPa, respectively. The high values of porosity, and therefore of absorption, reported for the CDW blocks could be attributed to the presence of FRA and CRA [38], since as mentioned above (Table 3), this mixture contained a 100% recycled aggregate as a substitute for a natural aggregate (FNA and CNA). In agreement with these results, Ren et al. [26] reported a direct relationship between the content of recycled aggregates (0–100% of AFR) and the absorption percentage (13–25%) of GFBS-based blocks. As such, the CDW block is proposed as an alternative material for the construction sector in Colombia, the use of which could meet the requirements established by the Ministry of the Environment and Sustainable Development (Resolution 0472 of 2017 [58]), requiring construction companies to use up to 30% recycled materials relative to the total weight of materials demanded by construction and civil engineering projects, regardless of their nature and/or type.

Regarding the RCBW blocks (Table 5), a compressive strength of 17.0 MPa was reported, which is 31% higher than the minimum strength (13 MPa) required by NTC 4026 [48] for the “high-strength structural” classification of blocks. The maximum allowable water absorption for this classification, considering the weight of the block (density 1536 kg/m^3^), is 15% (Table 4). The water absorption for the RCBW blocks was 11.2%. The porosity and modulus of rupture (flexural strength) were 17.2% and 1.5 MPa, respectively.

According to the specifications established by the NTC 4026 [48] standard (Table 4), the reference block (OPC) is classified as a “low-class structural” block. In this sense, it is highlighted that the alkali-activated blocks based on NP–GBFS, FA, and RCBW achieved a higher classification (high-strength structural) than that achieved by the OPC blocks.

The results of the physical-mechanical characterization of the blocks demonstrate the potential of application of alkali-activated materials in the production of prefabricated elements, suitable for use in the construction sector.

### 3.4. Design of the Eco-House Prototype

**Figure 5:**
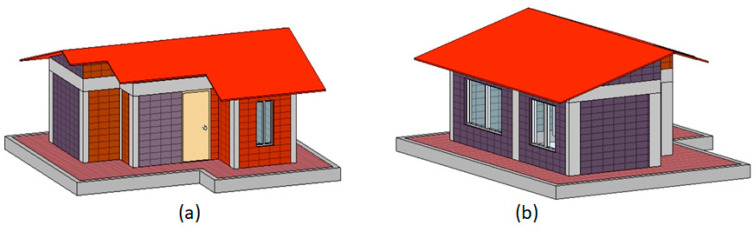
Architectural design (virtual modeling) of the eco-house prototype: (**a**) front view and (**b**) rear view.

The objective of this stage was to validate the potential of using these different alternative materials, applying the same construction methods and with the same performance requirements of traditional materials, taking into account that the construction of an eco-house from alkali-activated blocks has not been reported prior to this paper. Therefore, the architectural and structural design of the eco-house prototype (Figure 5) was established following the specifications of NSR-10 [50] for one- to two-story earthquake-resistant houses. 

The housing prototype had spaces representing the main room, integrated with a bathroom, kitchen, and living room, with a constructed area of 8.36 m^2^. The free height of the eco-house prototype was set at 1.3 m at the highest point (ridge). The windows were placed at the front and back of the house, considering ventilation and luminosity, which guaranteed thermal comfort and the use of natural light inside the house. The roof design was a “gabled” roof with slopes of 29% and 24% to facilitate the collection and use of water. Around the house, a platform was designed projecting 50 cm from each wall. Figure 5 shows the front facade (Figure 5a) and rear (Figure 5b) views of the eco-house according to the architectural design.

**Figure 6:**
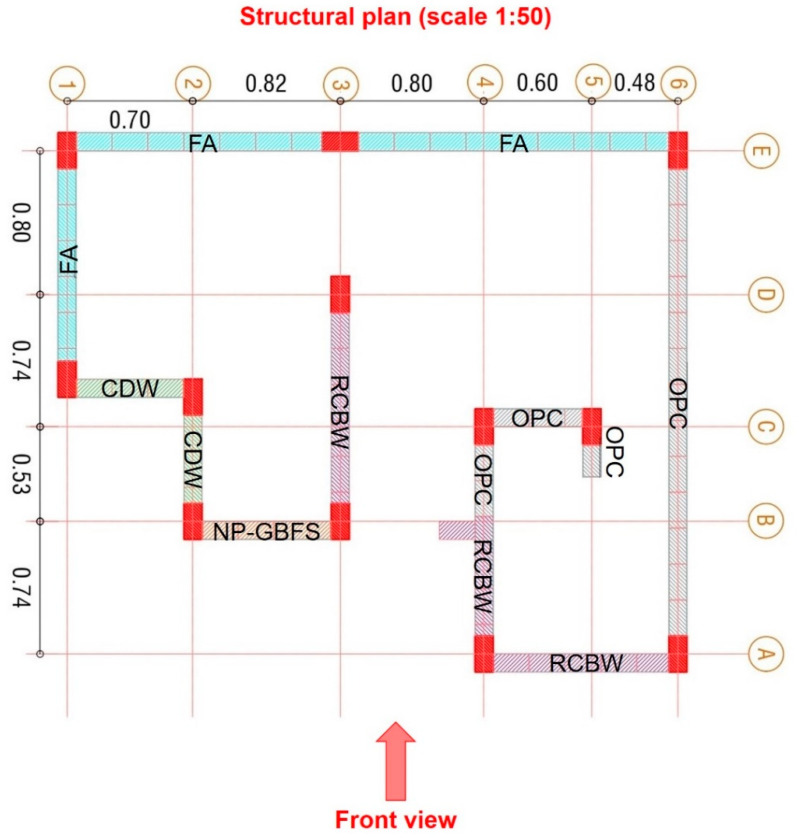
Structural plan of the eco-house prototype (1:50 scale) (distances in m) and definition of the type of block per wall.

Figure 6 presents a structural drawing (scale 1:50) of the eco-house prototype that defines the types of blocks used in the construction of each confined masonry wall and their dimensions. Note that for future comparative purposes (e.g., in thermal measurements, long-term and durability changes), some walls (axis 4 # B–C, axis 5 # C, axis C # 4–5, and axis 6 # A–E in the structural drawing) were built with reference blocks (OPC). 

The eco-house was designed around a seismic resistance system capable of guaranteeing an adequate response to vertical and horizontal loads. The foundation system was designed to guarantee the integral and balanced transmission of loads from the structure to the ground, with a system rigid enough to avoid differential settlement. The foundation was composed of a reticular system of continuous OPC concrete beams of 25 × 25 cm^2^. The longitudinal reinforcement consisted of four 12.7 mm diameter corrugated steel bars (rebar), reinforced with 9.5 mm diameter stirrups spaced 15 cm apart. The minimum concrete coverage was set at 50 mm. The foundation was cast on a 60-mm-thick hardfill layer. The concrete floor joist corresponded to rectangular OPC concrete beams of 10 × 20 cm^2^. The longitudinal reinforcement consisted of four corrugated steel bars of 9.5 mm in diameter, confined with 6.4 mm diameter stirrups spaced 15 cm apart.

For the design of the confined masonry walls, a wall thickness of 10 cm was established according to the dimensions of the blocks previously produced and a free height of 1.1 m. The blocks were confined by the OPC concrete columns and concrete tie beams. The confinement columns were designed with rectangular dimensions of 10 × 20 cm^2^ and a free height of 1.1 m. The reinforcing steel consisted of four longitudinal corrugated steel bars 9.5 mm in diameter, confined with stirrups 6.4 mm in diameter with 10 cm spacing. The reinforcement of the confinement columns was anchored in the lower part to the reinforced foundation and in the upper part to the reinforced concrete tie beams so that the monolithic behavior of the structure was guaranteed. The confinement beams corresponded to rectangular beams of 10 × 20 cm^2^. The reinforcing steel consisted of four longitudinal corrugated steel bars of 9.5 mm, confined by stirrups of 6.4 mm in diameter with a spacing of 10 cm. The reinforcement of the concrete tie beams (confinement) was anchored at the terminal ends with an angle of 90°. These rectangular reinforced concrete beams were arranged horizontally flush with the roof, forming closed rings to interlock the walls.

The concrete tie beams and lintels were OPC concrete elements with a square section of 10 × 10 cm^2^, reinforced with two longitudinal bars of corrugated steel of 9.5 mm in diameter, confined by helical (S-shaped) stirrups of 6.4 mm with a spacing of 15 cm. To guarantee the monolithic behavior of the structure, the reinforcement was anchored to the tie elements.

### 3.5. Construction of the Eco-House Prototype

**Figure 7:**
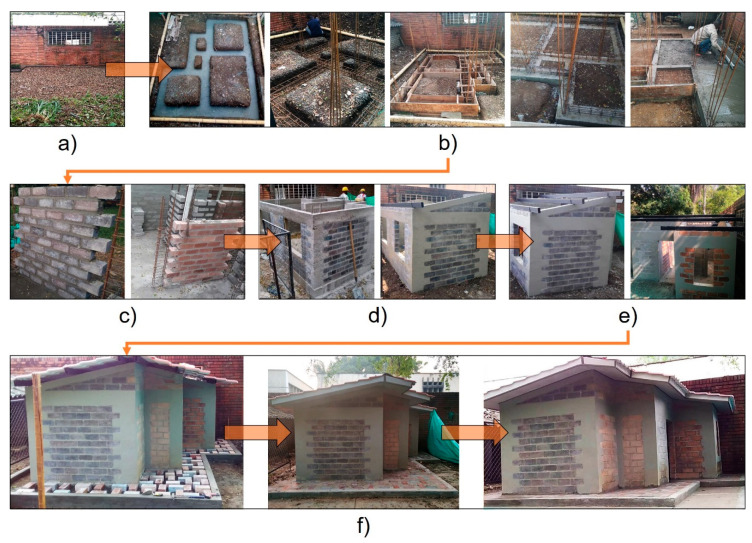
Construction process of the eco-house prototype: (**a**) adaptation of the terrain, (**b**) casting of concrete foundations and floor, (**c**) wall construction, (**d**) casting of confining columns and beams, (**e**) installation process of roof, and (**f**) construction of platforms and final finishing of the eco-house prototype.

Figure 7 presents the construction process of the eco-house prototype according to the structural and architectural design presented in Section 3.4. The construction of the eco-house prototype was carried out on the campus of the Universidad del Valle (Cali-Colombia), specifically in the back area of the School of Materials Engineering (building E44). Among the preliminaries of the construction, the adaptation of the terrain and the layout of the axes (Figure 7a) according to the structural drawing (Figure 6) and the provisions of section E.6.2.1 of NSR-10 [50] were implemented. Subsequently, the foundation was excavated, complying with the dimensions stipulated in the structural drawing. Once the excavation soil was removed from the bottom, the 60-mm-thick hardfill layer was cast according to section E.6.2.2 of NSR-10 [50].

Considering section E.6.2.3 of NSR-10 [50], the configuration of the steel that ties the reinforced column of the foundation beam was assembled. The reinforcement was installed over the hardfill, leaving 50 mm of free space on each side for concrete cover. Hooks bent at 90° were placed on the outer face of the terminal transverse elements, and 45 cm overlaps were placed on the longitudinal bars. Once the steel of the foundation was in place, the reinforced columns were fixed and anchored. It was verified that the layout of the steel met the specifications of the structural drawing, so the formwork was assembled and, finally, the beam was continuously cast with OPC concrete to guarantee a monolithic system. After the foundation cured, the concrete floor joist was configured and cast according to the design specifications. The rectangular spaces formed between the beams were filled with adequately compacted natural soil, leaving a free height of 70 mm at the zero level for the casting of the floor. A subfloor sheathing of 70-mm-thick concrete reinforced with an electro-welded mesh with square openings of 15 × 15 cm^2^ and a bar diameter of 4 mm was cast over the fill (Figure 7b).

The construction of the confined masonry walls was carried out according to the provisions of section E.6.3.1 of NSR-10 [50]. A total of 53 blocks of NP–GBFS, 165 blocks of FA, 120 blocks of CDW, 217 blocks of RCBW, and 303 blocks of OPC were used in the construction of the confined masonry walls. The blocks were previously moistened (pre-saturated) and set with a standard OPC mortar with a cement/sand ratio of 1:3 and 2 cm thick. Each wall was configured such that the vertical joints of the blocks were interlocked, leaving the space required to subsequently cast the confinement columns. At the lower limit of the windows, the OPC concrete lintels were cast according to the design specifications. After the lintels cured, the wall construction continued until the defined height was reached (Figure 7c).

Once the confined masonry walls were built, and after the curing process, the confinement columns and concrete tie beams of the reinforced OPC concrete were cast. The vertical reinforcement of the confinement columns was finished using 90° hooks that ran up to the top of the confinement beam, and a free space was left at the top of the 50 mm-coated hooks as a precaution. The casting of the OPC concrete in the confinement columns and concrete tie beams was performed continuously, allowing the concrete to contact the terminal surface of the confined wall (Figure 7d).

After the OPC concrete of the confinement elements cured, the masonry butt joints were built on the concrete tie beams using the same type of block selected for each wall in the structural design (Figure 6). On the blocks that formed the butt joints, concrete tie beams were cast according to the architectural design. On the concrete tie beams, rectangular steel structural beams with dimensions of 76 × 38 mm^2^ were placed that supported the installation of the covering or roof, as shown in Figure 7e. The roof was designed as a double-pitched roof with unplasticized polyvinyl chloride (UPVC) tiling. The tiles protruded from the edge of the walls with a horizontal projection of 30 cm.

Finally, for the construction of the platform, 10 × 20 cm^2^ rectangular concrete beams were cast in front of each wall, with a frontal separation of 50 cm from the concrete floor joist on each side. Above the platform internal space (50 cm wide), 14 NP–GBFS blocks, 8 FA blocks, 20 CDW blocks, 38 RCBW blocks, and 26 OPC blocks were randomly placed in the shape of a paver. A mixture of OPC concrete was poured between the openings or spaces left between the “pavers”. After the installation of the polyvinyl chloride (PVC) ceiling, an esthetic finish was applied to the construction, giving the appearance of the eco-house shown in Figure 7f.

### 3.6. Estimation of the Environmental Impact

The estimation of the environmental impact associated with the alkali-activated mixtures, from which the blocks were produced and with which the eco-house prototype was ultimately built, was performed using the global warming potential indicator (GWP). For the inventory analysis, the Ecoinvent 3.6 database was used [59]. The functional unit was 1 m^3^ of alkali-activated concrete mixture (NP–GBFS, FA, CDW, and RCBW), taking as reference the mixture designs that are reported in Table 3. The analysis of the carbon footprint of the alkali-activated mixtures was compared with that of the OPC-based reference mixture. Note that according to the yield of the concrete mixtures per cubic meter and the dimensions of the rectangular blocks (0.0016 m^3^ per unit), the number of blocks produced per cubic meter was approximately 625 units. 

Table 6 presents the CO_2_ emissions per kilogram (GWP: kg∙CO_2_∙eq) of each of the raw materials used in the production of the mixtures and blocks. According to these results, NaOH (1.46 × 10^0^ kg∙CO_2_∙eq), OPC (8.45 × 10^−1^ kg∙CO_2_∙eq), and Na_2_SiO_3_ (8.12 × 10^−1^ kg∙CO_2_∙eq) are, in that order, the raw materials with the highest CO_2_/kg emissions. Therefore, it is expected that the designs of mixtures with higher contents of these materials will have a larger carbon footprint; that is, the environmental impact of each concrete will ultimately depend on the design of the mixtures (the proportion of the materials (Table 3)). On the other hand, the precursors (NP, GBFS, FA, CDW, and RCBW) have a significantly lower GWP (kg∙CO_2_∙eq) than OPC (Table 6).

Regarding the high carbon footprint (CO_2_/kg) associated with alkaline activators (NaOH and Na_2_SiO_3_), these chemical reagents are based on natural raw materials and involve industrial processes with high energy costs and high CO_2_ emissions [60]. Sodium hydroxide (NaOH) is prepared mainly by electrolytic methods using an aqueous solution of sodium chloride. Sodium silicate (Na_2_SiO_3_) is initially obtained by mixing sodium carbonate (Na_2_CO_3_) and silica (SiO_2_). Then, the mixture is cast at a temperature range between 1100 and 1200 °C, producing an amorphous solid. The product is then introduced into an autoclave, subjected to high pressure, and, upon contact with water, yields an aqueous solution called “waterglass” [9]. In fact, the need for high temperatures to process sodium silicate substantially increases the carbon footprint of alkali-activated materials that incorporate this type of activator [61]. Therefore, one of the key aspects in alkaline activation technology is the synthesis of activators and sodium silicates based on alternative sources to produce more environmentally friendly alkali-activated materials [62,63,64].

**Figure 8:**
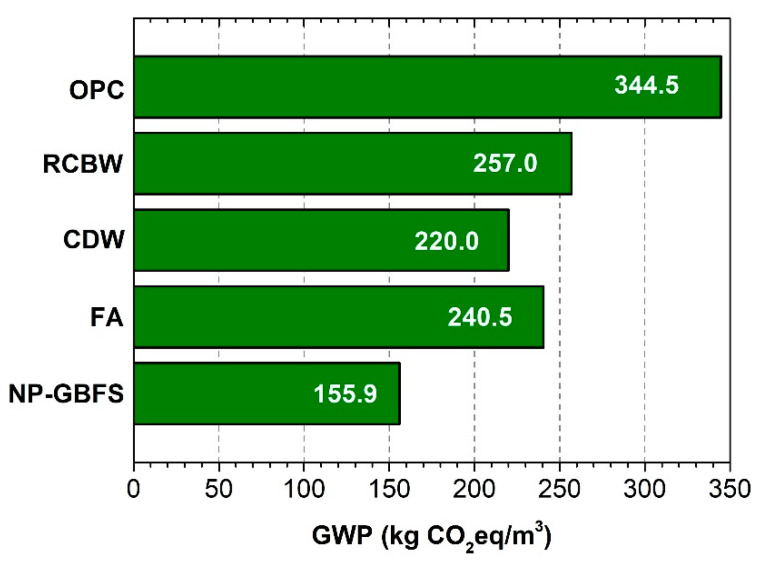
Carbon footprint (GWP: kg∙CO_2_∙eq/m^3^) associated with the raw materials of the concrete mixes used in the production of the blocks (alkali-activated blocks vs. OPC block).

Through the application of the emissions inventory (kg∙CO_2_∙eq) of the raw materials (Table 6) to the designs or proportions of mixtures (Table 3), the carbon footprint per cubic meter (kg∙CO_2_∙eq/m^3^) for each type of block associated with the raw materials was calculated. Note that the carbon footprint of the NP–GBFS, FA, CDW, and RCBW mixtures was 155.9, 240.5, 220, and 257 kg∙CO_2_∙eq/m^3^, respectively, in relation to the 344.5 kg∙CO_2_∙eq/m^3^ reported for the OPC mixture (Figure 8). The results, presented in Figure 8, represent an important finding in the environmental sustainability of the constructed eco-house prototype, since these GWP values are proportional to the carbon footprint of the blocks made and used in their construction. Reductions of 25.4–54.7% compared with the carbon footprint associated with the OPC-based reference blocks were achieved. As an approximation of the carbon footprint per unit (block) associated with the emissions of the raw materials, the GWP per cubic meter (kg∙CO_2_∙eq/m^3^) of each mixture was divided by 625 (the yield of 1 m^3^ of mixture represented in units of rectangular blocks of 0.0016 m^3^). This calculation yielded GWP values of 0.25, 0.38, 0.35, and 0.41 kg∙CO_2_∙eq/block for NP–GBFS, FA, CDW, and RCBW, respectively, in relation to the 0.55 kg∙CO_2_∙eq/block of the OPC block.

In Colombia, cargo transportation is generally carried out in trucks of 16 to 32 tons. Ecoinvent reports for this case a GWP of 1.72 × 10^−1^ kg∙CO_2_∙eq/km [59]. The distances to transport the raw materials to the block factory were estimated at 27.2 (OPC and GBFS), 26.8 (FA), 13.2 (RCD), 188.0 (NP), 20.0 (RCBW), 17.3 (activators), 37.2 (natural aggregates), and 17.0 km (recycled aggregates). Based on these data, it can be seen that the GWP value, included in Figure 8, increases from 11.1 (OPC concrete) to 46.4 (NP–GBFS concrete) kg∙CO_2_∙eq/m^3^. It should be noted that for the calculation, it has been considered that the block factory is located in the laboratory (campus of the Universidad del Valle), but at greater distances, the effect of transport can be significantly increased. In general, the effect on the GWP was 3% for the reference blocks, compared to the alkali-activated blocks, in which the increase was in the range of 5.5% to 23%. In conclusion, although the environmental impact associated with transport was not so significant compared to that of some of the mix components, it is important to clarify the need to make use of locally available materials to reduce the emissions generated in the transport of raw materials.

**Figure 9:**
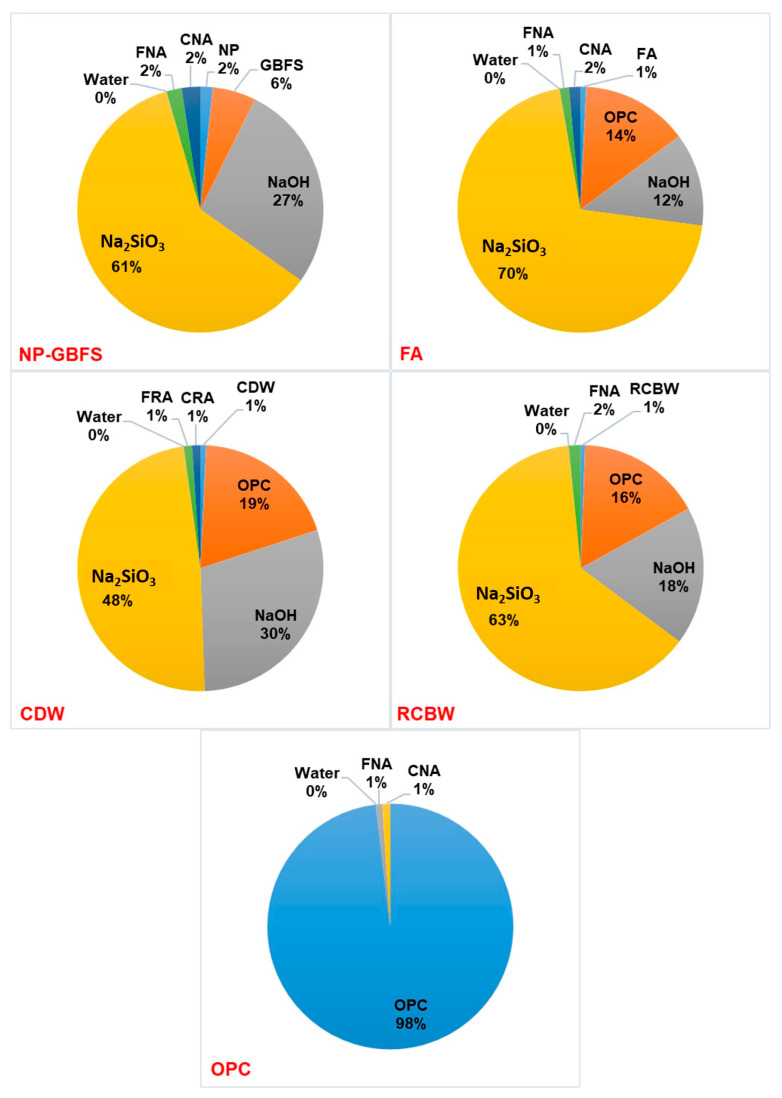
Contribution (%) of raw materials in the carbon footprint (GWP: kg∙CO_2_∙eq/m^3^) of the concrete mixes and/or blocks produced (alkali-activated blocks vs. OPC block).

Figure 9 shows the contribution (%) of each raw material to the GWP (Figure 8) of each of the concrete mixtures and/or block produced. As anticipated in the inventory of raw material emissions (Table 6), OPC and alkaline activators (NaOH and Na_2_SiO_3_) generate the most CO_2_ in mixtures and/or blocks. In the case of the alkali-activated blocks, the percentage contribution of the alkaline activators (NaOH and Na_2_SiO_3_) ranges between 77.8 and 88.2%, with the alkaline activators making the highest contribution to the total GWP for the NP–GBFS block. 

However, note that the NP–GBFS block has the highest environmental sustainability, yielding the lowest total GWP value (155.9 kg∙CO_2_∙eq/m^3^), because this block does not contain OPC as a calcium addition but instead, unlike the FA, CDW, and RCBW blocks, uses GBFS as a calcium source, which has a lower CO_2_/kg value (7.42 × 10^−2^) than the OPC (8.45 × 10^−1^) (Table 6). Note that authors such as Habert et al. [9], Komnitsas [7], and Scrivener et al. [65] reported that alkali-activated mixtures that incorporate GBFS have lower environmental impact because they require less alkaline activator, which is corroborated in the mixture designs reported in Table 3.

For the OPC block, Portland cement contributes 98.1% (338 kg∙CO_2_∙eq) of the total emissions (344.5 kg∙CO_2_∙eq/m^3^). In contrast, for the FA, CDW, and RCBW blocks that use OPC as a calcium source, the contribution of OPC in the total carbon footprint ranges between 14 and 19%, demonstrating the benefit of hybrid alkali-activated mixtures with low OPC content (≈10%). 

## 4. Conclusions

This article demonstrated the application potential of alkali-activated materials in the production of blocks for the construction of environmentally sustainable housing, complying with all the physical-mechanical specifications established by the technical standards and construction regulations that guarantee their structural performance and earthquake (or seismic) resistance. The eco-house prototype represents an interesting and high-potential object of future studies that will be related to thermal measurements (comfort) and the long-term durability of the alkali-activated blocks.

From the experimental results and their respective analysis, the following conclusions can be drawn:

- Alkali activation technology or geopolymerization is a sustainable method that uses various types of aluminosilicates (precursors), including natural pozzolans (NP), granulated blast furnace slag (GBFS), fly ash (FA), construction and demolition waste (CDW) (concrete, ceramics, mortar, and bricks wastes), and red clay brick waste (RCBW). Note that the latter are industrial by-products or waste, considered worldwide as an environmental problem due to their extensive generation and poor management. In this sense, alkaline activation from the use of this type of waste is proposed as a comprehensive alternative and an approach towards the implementation of the “circular economy” in the construction sector.
- The alkali-activated blocks produced met all the physical-mechanical specifications established by the Colombian Technical Standard (NTC) to be classified as concrete units for structural masonry. The compressive strengths (28 days) of the NP–GBFS (31.4 MPa), FA (23.9 MPa), CDW (26.1 MPa), and RCBW (17.0 MPa) blocks and the reference OPC block (22.7 MPa) exceeded the minimum limit established by NTC 4026 (equivalent to ASTM C90) for its structural classification (low class ≥ 8 MPa and high class ≥ 13 MPa).
- The architectural and structural design, as well as the construction process, of the eco-house prototype met all the specifications of the Colombian Regulations for Earthquake Resistant Buildings (NSR-10) for one- and two-story houses.
- The results obtained show that NaOH (1.46 × 10^0^ kg∙CO_2_∙eq), OPC (8.45 × 10^−1^ kg∙CO_2_∙eq), and Na_2_SiO_3_ (8.12 × 10^−1^ kg∙CO_2_∙eq) are, in that order, the raw materials with the highest CO_2_/kg emissions among all the raw materials used. However, the carbon footprint of alkali-activated blocks ultimately depends on the design of the mixtures or the proportions of these materials. In this sense, the alkali-activated blocks had carbon footprints 25.4–54.7% lower than that of the OPC-based reference blocks. In effect, the global warming potential (GWP) values of the NP–GBFS, FA, CDW, and RCBW mixtures were 155.9, 240.5, 220.0, and 257.0 kg∙CO_2_∙eq/m^3^, respectively, compared to the GWP of 344.5 kg∙CO_2_∙eq/m^3^ of the OPC mixture. Regarding the masonry units (625 concrete blocks/m^3^), the GWP values associated with the raw materials of the NP–GBFS, FA, CDW, and RCBW blocks were 0.25, 0.38, 0.35, and 0.41 kg∙CO_2_∙eq/block, respectively, compared with the 0.55 kg∙CO_2_∙eq/block produced by the OPC block. By including the transportation of raw materials in the GWP calculation, although the values increase up to 23%, the total GWP of alkali-activated blocks was lower than that of the reference blocks.
- In summary, the study shows the feasibility of making use of industrial by-products and wastes (GBFS, FA, RCBW, RCD) as raw materials to produce alkali-activated blocks with a low environmental footprint and appropriate characteristics for the construction of houses, complying with the specifications of the construction codes. This option is in accordance with the principles of the circular economy. Future studies should focus on evaluating the thermal performance and durability of the prototype.

## Figures and Tables

**Table 1:** Chemical composition (XRF) of the raw materials (construction and demolition waste (CDW) and ordinary Portland cement (OPC)).

Material	SiO_2_	Al_2_O_3_	Fe_2_O_3_	CaO	Na_2_O	MgO	K_2_O	LOI	SiO_2_/Al_2_O_3_ Molar Ratio	Particle Size (µm)	Density (kg/m^3^)
NP	62.0	15.5	7.3	5.2	4.1	2.5	1.6	0.5	6.8	20.6	2780
GBFS	37.7	15.7	1.8	40.3	0.2	1.3	0.4	-	4.1	26.4	2918
FA	59.0	23.9	5.9	0.74	0.2	0.3	1.2	6.3	4.2	24.9	2396
CDW	47.6	11.2	5.9	21.2	0.6	1.1	1.1	9.1	7.2	92.1	2690
RCBW	65.9	20.1	9.1	0.7	0.4	0.9	1.0	-	5.6	24.2	2560
OPC	17.9	3.9	4.8	62.3	0.2	1.8	0.3	4.1	-	21.6	3121

LOI: loss on ignition.

**Table 2:** Characteristics of the aggregates (natural and recycled) used in the production of the blocks.

Characteristics	Standard	FNA	FRA	CNA	CRA
Density (kg/m^3^)	ASTM C127 [42]	2580	2029	2540	2326
Absorption (%)	ASTM C128 [43]	1.9	12.1	2.0	9.2
Unit weight (kg/m^3^)	ASTM C29 [44]	1630	1240	1470	1211
Maximum size (mm)	ASTM C136 [45]	N/A		25.4	25.4
Fineness modulus		2.6	3.0	N/A	
Resistance to degradation, %	ASTM C131 [46]	N/A		16.4	33.6

**Table 3:** Proportion of the mixtures (kg/m^3^) used to produce the blocks.

Material	Types of Mixes				
	NP–GBFS	FA	CDW	RCBW	OPC
NP	280.0	-	-	-	-
GBFS	120.0	-	-	-	-
FA	-	360.0	-	-	-
CDW	-	-	450.0	-	-
RCBW	-	-	-	450.0	-
OPC	-	40.0	50.0	50.0	400.0
NaOH	29.3	20.2	44.4	32.0	-
Na_2_SiO_3_	116.6	207.8	131.0	200.0	-
Water	103	60.8	151.2	100.0	260.0
FNA	761.6	722.6	-	1000.0	707.8
FRA	-	-	604.8	-	-
CNA	930.9	883.2	-	-	865.1
CRA	-	-	604.8	-	-
Total (kg/m^3^)	2341.4	2294.6	2036.2	1832.0	2232.8

**Table 4:** Compressive strength and water absorption requirements, and classification by weight for structural masonry concrete units, according to NTC 4026 [48] (equivalent to ASTM C90 [49]) and NSR-10 [50] title E.

Class	Minimum Compressive Strength (28 days)	Maximum Water Absorption (%) According to the Weight (Density) (kg/m^3^)		
		Low Weight (<1680 kg/m^3^)	Medium Weight (1680–2000 kg/m^3^)	Normal Weight (≥2000 kg/m^3^)
High	13	15	12	9
Low	8	18	15	12

**Table 5:** Characteristics and properties of the produced blocks (alkali-activated and OPC blocks).

Properties (28 days)	Type of Block				
	Alkali-Activated				OPC
	NP–GBFS	FA	CDW	RCBW	
Compressive strength (MPa)	31.4	23.9	26.1	17.0	22.7
Modulus of rupture (MPa)	4.8	4.6	3.6	1.5	5.2
Density (kg/m^3^)	1902.2	2295.2	1925.8	1535.8	2084.5
Absorption (%)	8.3	7.5	14.4	11.2	10.6
Porosity (%)	15.6	17.2	27.7	17.2	22.2
Structural block class	High	High	Low	High	Low

**Table 6:** Emissions inventory (global warming potential (GWP): kg∙CO_2_∙eq) of the raw materials used in the production of the blocks. Data source: Ecoinvent 3.6 [59].

Raw Materials		GWP (kg∙CO_2_∙eq)
Cement	OPC	8.45 × 10^−1^
Precursors	NP	9.10 × 10^−3^
	GBFS	7.42 × 10^−2^
	FA *	5.26 × 10^−3^
	CDW	3.80 × 10^−3^
	RCBW	3.27 × 10^−3^
Alkaline Activators	NaOH	1.46 × 10^0^
	Na_2_SiO_3_	8.12 × 10^−1^
Water	H_2_O	2.10 × 10^−4^
Fine Aggregates	FNA	4.11 × 10^−3^
	FRA	3.98 × 10^−3^
Coarse Aggregates	CNA	4.11 × 10^−3^
	CRA	3.98 × 10^−3^

* Data source: Habert et al., 2011 [9].

## Data Availability

Data sharing not applicable.
